# Extreme levels of fallout radionuclides and other contaminants in glacial sediment (cryoconite) and implications for downstream aquatic ecosystems

**DOI:** 10.1038/s41598-019-48873-z

**Published:** 2019-08-29

**Authors:** Philip N. Owens, William H. Blake, Geoffrey E. Millward

**Affiliations:** 1 Environmental Sciences Program and Quesnel River Research Centre, University of Northern British Columbia, Prince George, British Columbia, V2N4Z9 Canada; 2 School of Geography, Earth and Environmental Sciences, University of Plymouth, Plymouth, Devon UK

**Keywords:** Biogeochemistry, Environmental chemistry, Hydrology

## Abstract

Glaciers in most parts of the world are retreating, releasing water and sediments to downstream rivers. Studies have found elevated levels of fallout radionuclides (FRNs) and other contaminants in glacial sediments, especially cryoconite, in European glaciers and Greenland. However, there are no equivalent studies for glaciers in North America. We report concentrations of FRNs (i.e. ^137^Cs, ^210^Pb_un_ and ^241^Am) and other contaminants (i.e. metal(loids), phosphorus) in cryoconite and proglacial sediments from a glacier in British Columbia, Canada, and compare values to suspended sediments from the downstream river. The mean concentrations of ^137^Cs, ^210^Pb_un_ and ^241^Am in cryoconite were 2,123 ± 74, 7,535 ± 224 and 11.5 ± 3.0 Bq kg^−1^, respectively, which are an order of magnitude greater than those for most soils and surficial materials. FRNs were much lower in suspended sediments and decreased with distance away from the glacier. Geochemical elements were enriched in cryoconite relative to local clastic materials and upper continental crust. Concentrations of FRNs in cryoconite were correlated with organic matter, which suggests this is important in controlling the scavenging of hydrophobic contaminants in glacial meltwater. Low concentrations of FRNs and contaminants in suspended sediments suggest that glacial meltwater and the delivery of cryoconite have limited impact on downstream aquatic ecosystems.

## Introduction

It is well documented that glaciers in most parts of the world are retreating at a rapid rate due to global warming. In western Canada, for example, it is estimated that most glaciers will have vanished by 2100^1^. Consequently, rivers that drain glacierized areas are experiencing changes in water flows and sediment fluxes^2^; the latter reflecting a combination of increased water flows and sediment availability due to the exposure of new sources. Studies^3,4^ have reported that glaciers and snowpacks in mountain areas, such as the Canadian Rocky Mountains and the European Alps, contain contaminants including persistent organic pollutants (POPs), which are derived from remote sources and are transported and deposited on the surfaces of glaciers by atmospheric processes. For example, elevated activities of artificial fallout radionuclides (FRNs) such as caesium-137 (^137^Cs) and americium-241 (^241^Am), as well as the natural fallout product unsupported lead-210 (^210^Pb_un_), have been found in supraglacial cryoconite sediments in the European Alps^5^. Cryoconite is a specific type of supraglacial sediment found on the surface of glaciers, typically in the ablation zone and is often found in holes caused by melting due to the thermal properties of the dark sediment and impurities in the ice. Cryoconite holes are found in a variety of glacial environments, including Antarctica^6^. The particulate material is composed of a combination of atmospheric dust derived from regional and distal sources, minerogenic sediment from adjacent slopes, and organic material including bacteria and other organisms^7^. The inclusion of a significant proportion of organic material separates cryoconite from other types of supraglacial material, and organic matter contents are typically between 5 and 10% and can be up to ~20%^7^.

The on-going melting of glacial ice is likely to free contaminants contained within cryoconite holes for release to downstream rivers, which in turn may affect aquatic ecosystems and human health. Studies^8^ have already shown elevated levels of polychlorinated biphenyls (PCBs) and other contaminants in downstream aquatic systems due to melting glaciers. However, our understanding of the role that cryoconite plays in absorbing contaminants and regulating fluxes of contaminants to downstream aquatic systems is still poor and requires “urgent attention”^7^, given the effects of climate change on current and future glacier mass balances.

**Figure 1:**
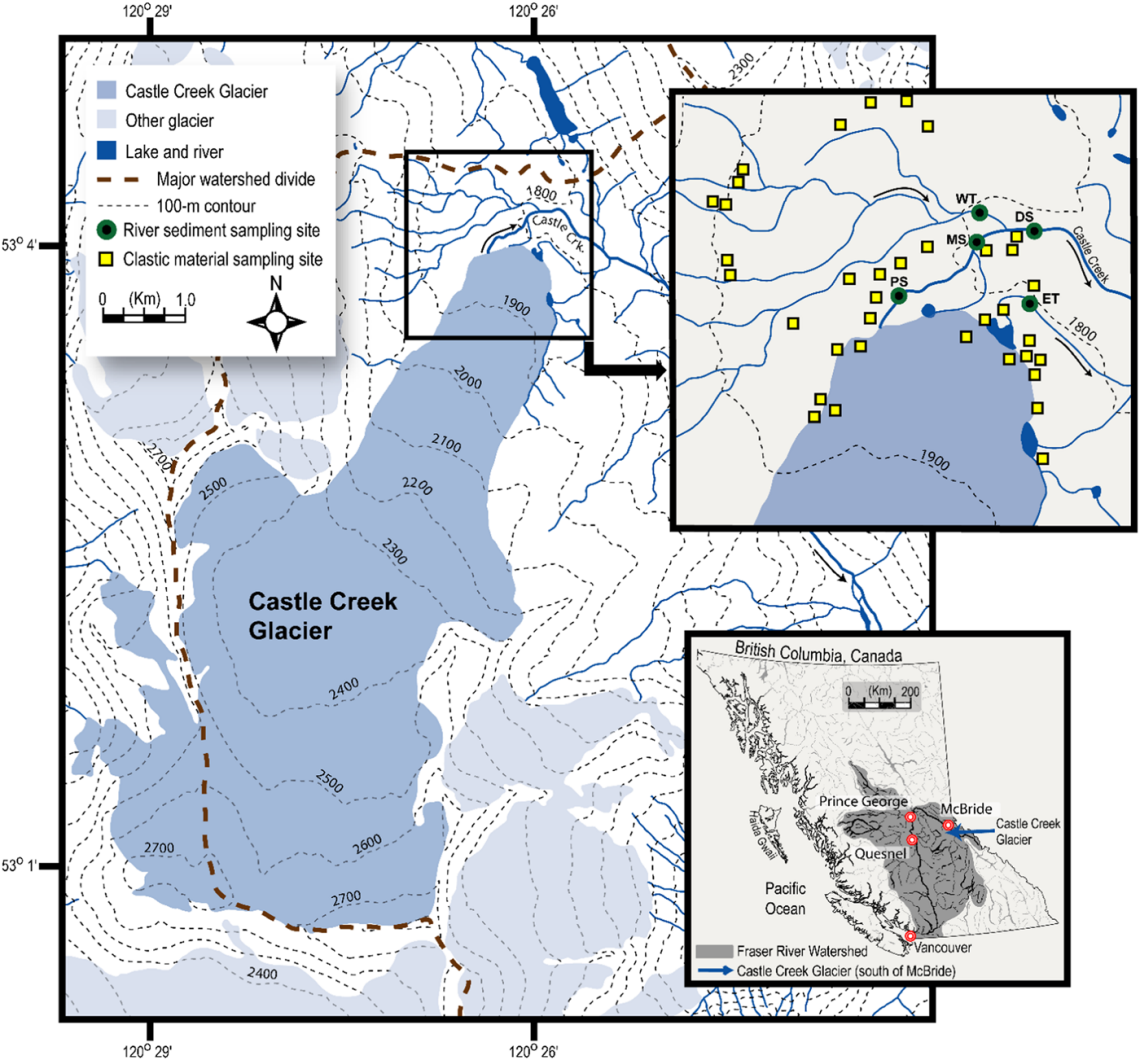
Location of Castle Creek Glacier, British Columbia, Canada. Top inset map shows the location of cryoconite and other clastic materials (subglacial, proglacial, talus and channel bank) and river sediment sampling sites: PS = Proximal Site; MS = Middle Site; DS = Distal Site; WT = West Tributary; ET = East Tributary. Sites PS, MS and DS are on the main proglacial river. Multiple samples were collected at some locations.

Despite several recent studies documenting elevated levels of FRNs in cryoconite in glaciers from Europe^5,9–17^, as far as we are aware no studies on elevated fallout contaminants in cryoconite have been conducted in North America. Furthermore, few studies have compared FRN concentrations in cryoconite to concentrations in fluvial sediments being actively transported in downstream proglacial rivers, to assess the broader environmental and ecological significance. In this context, we report the activities of ^137^Cs, ^210^Pb_un_ and ^241^Am and the concentrations of other contaminants, such as trace elements, metal(loids) and phosphorus, in cryoconite and proglacial sediments from a glacier in British Columbia, Canada (Fig. 1). Unlike most parts of Europe, the study area is essentially free of Chernobyl fallout, although there are regional sources of atmospheric radioactive contamination such as the Hanford Nuclear Site in Washington State (46° 39′N, 119° 36′W) and Nevada Test Site (37° 07′N, 116° 03′W), both in the USA. Specific objectives were: 1) to determine whether the FRNs and geochemical elements in cryoconite had elevated concentrations relative to local non-glacial sources; and 2) to assess if the fluvial sediments in the receiving proglacial river had elevated concentrations of contaminants and thus posed a risk to aquatic ecosystems.

## Results

### Activity concentrations of fallout radionuclides

**Figure 2:**
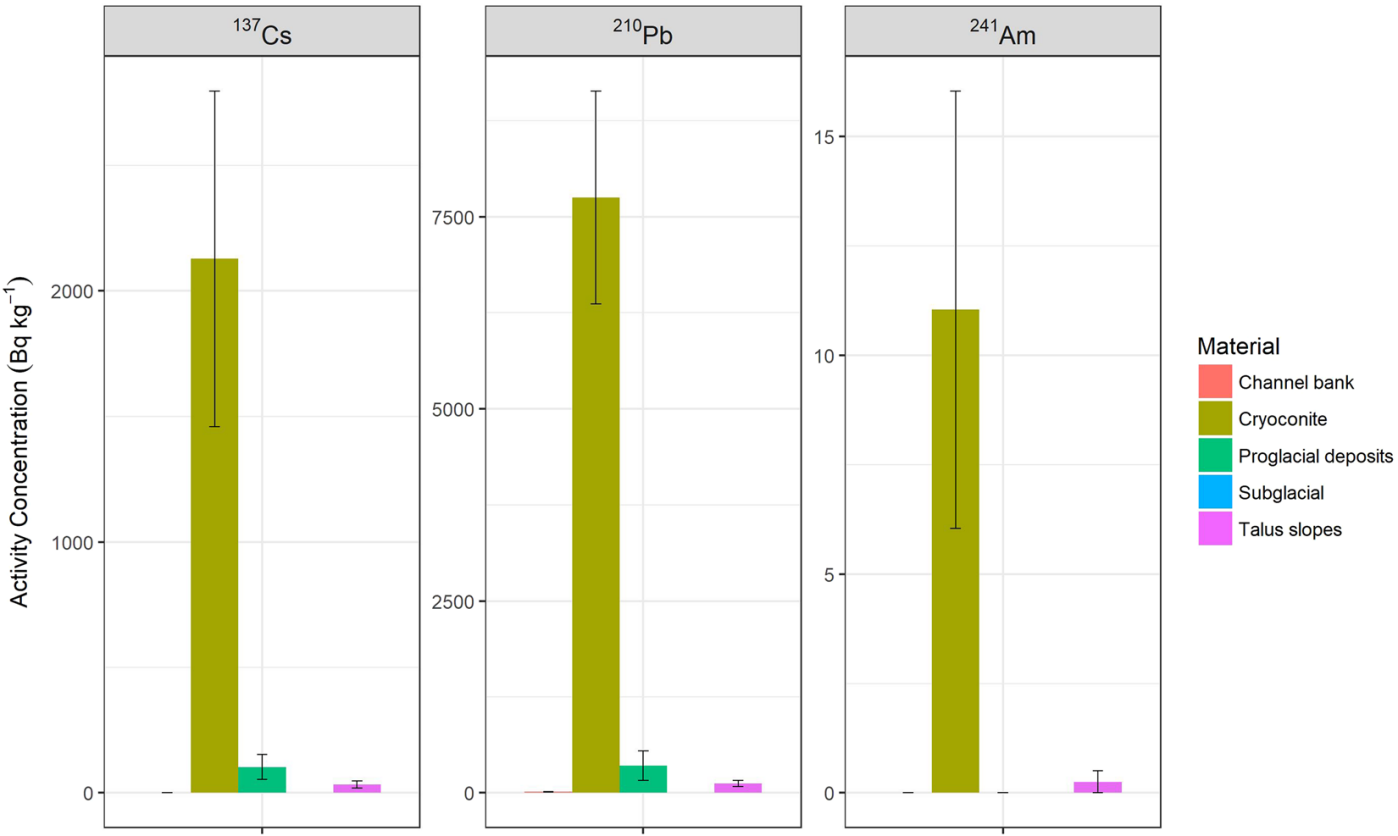
Fallout radionuclide activity concentrations (average ± 1 SD; Bq kg^−1^ dry weight) for ^137^Cs, unsupported ^210^Pb, and ^241^Am in cryoconite samples from Castle Creek Glacier and other clastic materials.

The average (±1 standard deviation, SD) activity concentrations of ^137^Cs, ^210^Pb_un_ and ^241^Am in cryoconite were 2,123 ± 74, 7,535 ± 224 and 11.5 ± 3.0 Bq kg^−1^, respectively (Fig. 2); the highest activities for an individual sample were 3,969 ± 149, 11,642 ± 456 and 25.6 ± 4.1 Bq kg^−1^, respectively. In contrast, the activity concentrations for the subglacial sample were below minimum detectable activity (MDA; <3.9, <38.7 and <3.9 Bq kg^−1^, respectively) for all three FRNs. Values for the proglacial sediment collected near to the glacier snout had much lower FRN activity concentrations than cryoconite; 101 ± 4.8, 354 ± 40 and <2.9 Bq kg^−1^ for ^137^Cs, ^210^Pb_un_ and ^241^Am, respectively. Activity concentrations for (i) the talus material collected from slopes away from the glacier, and thus not expected to be influenced by glacial ice, and (ii) material collected from eroding channel banks along the main proglacial channel, had much lower values for ^137^Cs and ^210^Pb_un_ (Fig. 2) and values for ^241^Am were below detection (<3.0 and <2.6 Bq kg^−1^, respectively).

Activity concentrations of FRNs in fluvial suspended sediments from the main proglacial stream had maximum values closest to the glacier, which decreased with increasing distance from the glacier such that the lowest values were at ~1 km from the glacier snout (Table 1). Values of FRNs for samples from a small tributary stream draining the slopes in the unglacierized part of the watershed were all <MDA.

**Table 1:** Average fallout radionuclide activity concentrations (±1 SD; Bq kg^−1^ dry weight) in suspended sediment samples (n = 2 for each site) from the main proglacial stream (at increasing distance from the glacier snout) and several tributaries.

Location	Activity concentrations (Bq kg^−1^)		
	^137^Cs	^210^Pb_un_	^241^Am
Main stream: proximal	25 ± 6	128 ± 122	<1.9
Main stream: middle	14 ± 1	62 ± 22	<1.7
Main stream: distal	10 ± 2	32 ± 22	<2.2
East tributary (glacier)	16 ± 4	27 ± 37	<1.8
West tributary (slopes)	<1.5	<19	<1.9

The West tributary does not receive meltwater from the glacier.

### Geochemical elements

**Figure 3:**
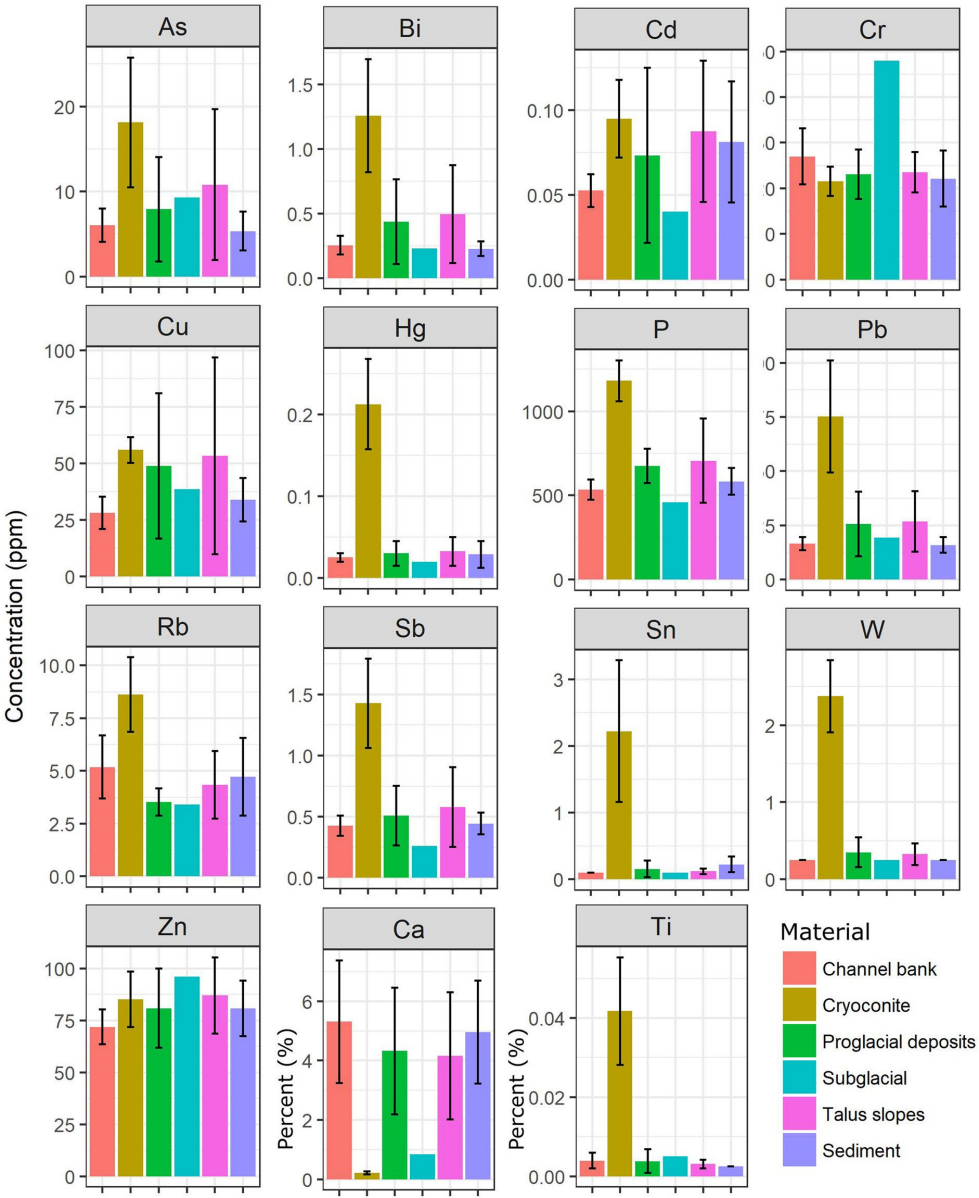
Concentrations (average ± 1 SD) of geochemical elements in cryoconite and other local source materials and suspended sediments collected from the Castle Creek Glacier study area.

Concentrations for elements that have been identified as being elevated in cryoconite in other studies^5,9,12,18,19^ are presented in Fig. 3. The degree of elemental enrichment was determined using enrichment ratios (ER), calculated by comparing element concentrations for cryoconite samples to equivalent values for: (i) the average upper continental crust (ER_UCC_; based on published values^20,21^); and (ii) combined talus and channel bank materials from the local Castle Creek Glacier proglacial zone (ER_L_). In the case of the latter, both sets of concentrations were normalized relative to the concentrations of Al for each material type. The advantage of ER_L_ compared to ER_UCC_ is that the former provides a measure of local enrichment using similar clastic materials.

Several geochemical elements were elevated in cryoconite (Fig. 3) compared to other materials with ER_L_ values ranging from >2 (e.g. As, Bi, Hg, P, Pb, Rb, Sb, Ti and W) to 25.8 (Sn) (Table 2). Metals such as Cd, Cr, Cu and Zn (Fig. 3) were only slightly elevated in cryoconite relative to other materials with ER_L_ between 1 and 2 (Table 2). In the case of Ca, concentrations in cryoconite were low (ER_L_ = 0.06) compared to other local clastic materials. For the ER_UCC_ values, cryoconite samples from Castle Creek Glacier are moderately to highly enriched relative to average upper continental crust for As, Bi, Cu, Hg, and Pb (all >2), slightly enriched (between 1 and 2) for Cd, Sb, Sn and Zn, and depleted (<1) for Ca, Cr, P, Rb, Ti and W (Table 2).

**Table 2:** The enrichment ratios for the cryoconite samples estimated using local talus and channel bank material (ER_L_) and average upper continental crust (ER_UCC_) data.

	Enrichment ratios and sediment quality guidelines														
	As	Bi	Ca	Cd	Cr	Cu	Hg	P	Pb	Rb	Sb	Sn	Ti	W	Zn
ER_L_	2.71	4.21	0.06	1.71	1.10	1.73	9.40	2.42	4.39	2.34	3.61	25.8	15.4	10.4	1.37
ER_UCC_	3.18	5.48	0.09	1.58	0.29	2.07	4.25	0.24	4.43	0.09	1.90	1.01	0.12	0.17	1.14
SQG PEL^31,32^	17			3.5	90	197	0.49		91.3						315
SQG CBPEC^33^	33			4.98	111	149	1.06		128						459

Sediment quality guidelines (SQG; µg g^−1^) for freshwater aquatic life are also given for the probable effects level (PEL) and consensus-based probable effects concentration (CBPEC).

Element concentrations for suspended sediment samples were typically more similar to values for talus and channel bank materials than cryoconite (Fig. 3) and were also similar to values typically reported for upper continental crust^20,21^.

## Discussion

### Fallout radionuclides distribution between sources and sediments

The activity concentrations of FRNs for cryoconite from Castle Creek Glacier are highly elevated compared to values reported for most soils and sediments, except in areas influenced by nuclear plant incidents such as Chernobyl (Ukraine, April 1986) and Fukushima (Japan, March 2011). Activity concentrations for ^137^Cs and ^241^Am in natural soils and sediments are typically within the range 0.5–600 and 0.05–3 Bq kg^−1^, respectively^5^. The values for the other sediment types – proglacial, talus, channel bank and subglacial – fall within the ranges for typical environmental samples. However, the proglacial sediment collected in the immediate vicinity of the glacier snout had activity concentrations that were closer to values for cryoconite, which indicates that such material has input from cryoconite released from melting ice in addition to sediment from other sources such as subglacial and talus.

The FRN activity concentrations for cryoconite samples from Castle Creek Glacier are of the same order as values recently reported for cryoconite collected from the Morteratsch Glacier in the Swiss Alps^5^, with average (±1 SD) values of 2,700 ± 380 and 30 ± 35 Bq kg^−1^ for ^137^Cs and ^241^Am, respectively; for unsupported ^210^Pb the average value is ~2,800 Bq kg^−1^ (Table 3). These activity concentrations are also comparable to values reported for glaciers in Svalbard and the Italian Alps. The FRN activity concentrations for cryoconite from Castle Creek Glacier and several other glacier sites in Table 3 (i.e. Greenland, Svalbard and Switzerland) are considerably lower than values reported by Tieber *et al*.^10^ for small glaciers in the Austrian Alps, where values for 11 cryoconite samples range from 1,744 ± 150 to 140,051 ± 8,460 Bq kg^−1^ for ^137^Cs and <2.2 (no SD provided) to 93.3 ± 9.4 Bq kg^−1^ (decay corrected to 2006) for ^241^Am (also see Wilflinger *et al*.^16^). These extreme values likely reflect a combination of bomb-derived fallout and inputs from the Chernobyl incident in 1986.

**Table 3:** Fallout radionuclide activity concentrations in cryoconite for Castle Creek Glacier compared to other studies. Values are average ±1 SD or the range with the measurement error.

Locations	Approximate Coordinates	Activity Concentrations, Bq kg^−1^			Reference
		^137^Cs	^210^Pb	^241^Am	
Castle Creek Glacier, Canada	55°N; 120°W	2,123 ± 74	7,535 ± 224	12 ± 3	This study
Mittivakkat Glacier, Greenland	65°N; 50°W	231 ± 170	—	—	17
Morteratsch Glacier, Switzerland	46°N; 10°E	2,700 ± 380	2,800	30 ± 35	5
Glaciers in Italian Alps, Italy	46°N; 8°E	2,446 ± 36 to 8,894 ± 159	—	13.7 ± 6.7 to 40.8 ± 12.8	11
Stubacher Sonnblickkees, Austria^a^	48°N; 16°E	357 ± 43.3 to 223,150 ± 7,100	1,590 ± 76 to 57,500 ± 2,300	MDA to 70.3 ± 8.6	16
Glaciers in Austrian Alps, Austria^a^	48°N; 16°E	1,744 ± 150 to 140,051 ± 8,460	—	<2.2 to 93.3 ± 9.4	10
Scott Glacier, Svalbard	77°N; 15°E	285	483	—	9
Hans Glacier, Svalbard	77°N; 15°E	356 ± 58	2,335 ± 156	—	12
Werenskiold Glacier, Svalbard	77°N; 15°E	700 to 4,500	4,000 to 9,500	—	13
Adishi Glacier, Georgia	43°N; 43°E	580 ± 180 to 4,940 ± 610	1,400 ± 100 to 12,000 ± 600	8.1 ± 0.4 to 68.3 ± 4.3	14
**Potential Recent Sources**					
Hanford Test Site	46°N; 119°W				
Chernobyl	51°N; 30°E				
Novaya Zamlya	75°N; 55°E				
Fukushima	37°N; 141°E				

^a^Values also reflect Chernobyl fallout.

The FRN activity concentrations for fluvial sediment samples from the proglacial stream draining the glacier are much lower than the values for the cryoconite, and values decrease with increasing distance from the glacier snout. This likely reflects inputs of sediment from other sources in the proglacial zone with lower FRN concentrations, which dilute the high FRN concentrations associated with the cryoconite. Monitoring of suspended sediment fluxes at several locations along the Castle Creek Glacier proglacial stream in 2011^22^ showed that sediment sources derived from the glacier and proximal zone decreased during high-flow events, and that other sediment sources such as inputs from the slope and channel banks along the valley sandur became more important. The low FRN activity concentrations (<MDA for all FRNs) in the sediment samples collected from the tributary stream draining the non-glacierized slopes (i.e. West tributary, Table 1) further emphasizes the role of glacial meltwater in explaining the high FRN concentrations in the cryoconite and also the proglacial material (Fig. 2) and suspended sediment (i.e. proximal site, Table 1) close to the glacier snout.

### Geochemical elements distribution between sources and sediments

For many minor and major geochemical elements, concentrations in cryoconite are elevated compared to local clastic materials (e.g. talus and channel bank) and average values for upper continental crust. Other studies have also reported similar findings for Pb^8,11^ and As, Hg and Sb^5^. At Castle Creek Glacier the highest local ERs were for Sn, Ti and W. The relatively high values found in cryoconite might reflect atmospheric deposition associated with regional sources. There are numerous metal mines and other forms of natural resource extraction in British Columbia. The mines (both historic and active) have extracted numerous metals including Ag, Au, Bi, Cu, Hg, Mo, Pb, W and Zn, and several of these have associated by-products like As and Se, and also P from apatite-rich rocks. While numerous studies have documented pollution of soils and riverine sediments associated with these mines^23–25^, there is less information available on atmospheric deposition of these anthropically derived elements in central British Columbia. However, this does not explain the high enrichment ratios as talus and other local materials would also be expected to have similar high concentrations if such values only reflected rates of atmospheric deposition. Instead, it may reflect the high scavenging by clays, organic matter and bacteria within the cryoconite, which is discussed below.

Similar to the pattern for the FRNs, element concentrations in the suspended sediment being actively transported in the proglacial stream are consistent with values for non-glacial sources such as talus and channel bank material (Fig. 3). This suggests that these materials are the main source of the suspended sediment in the proglacial stream, and not the cryoconite. In line with data reported elsewhere^5^, Ca in cryconite was depleted relative to the other local materials, probably as Ca is fairly soluble and thus flowing water likely desorbs Ca out of the cryoconite. This may reflect the low to neutral pH of meltwaters and cryoconite porewaters.

For all materials, including cryoconite and suspended sediment, metal(loid) concentrations were below upper threshold sediment quality guideline (SQG) values for the protection of freshwater aquatic ecosystems (Table 2). Only As concentrations for cryoconite were slightly above the probable effects level (PEL) for Canada but were below the consensus-based probable effects concentration (CBPEC) which is obtained from multiple SQGs and thus is more representative of a wider range of environments.

### The role of cryoconite in scavenging elements from meltwater

Samples of cryoconite collected from Castle Creek Glacier have elevated levels of FRNs that are orders of magnitude higher than local clastic materials and suspended sediments. While deposition of atmospheric dust particles onto the glacier surface, which are enriched in FRNs relative to most other materials such as soils, may partly explain this phenomenon^10^ it is likely that scavenging by cryoconite of colloidal and dissolved materials in passing meltwater is the main process^5,12^. Cryoconite is typically composed of fine minerogenic particles (e.g. clays and silts) and organic material (including extracellular polymeric substances, EPS) which have high binding capacities for FRNs, trace elements and metal(loids), and nutrients like P^26–28^. During the ablation season, large quantities of water derived from melting snow and ice flow across the glacier surface to the ablation zone of the glacier, where much of the cryoconite is located, often in cryoconite holes. Contaminants previously locked-up within the snow and ice are released and subsequently sorbed and immobilized by the cryoconite. The degree of sorption, and thus enrichment of contaminants, reflects their affinity to bind to clays (e.g. ^137^Cs) and/or organic material (e.g. ^210^Pb_un_, Hg). Thus, given increases in glacial melt in recent decades, cryoconite is experiencing contact with larger quantities of meltwater.

**Figure 4:**
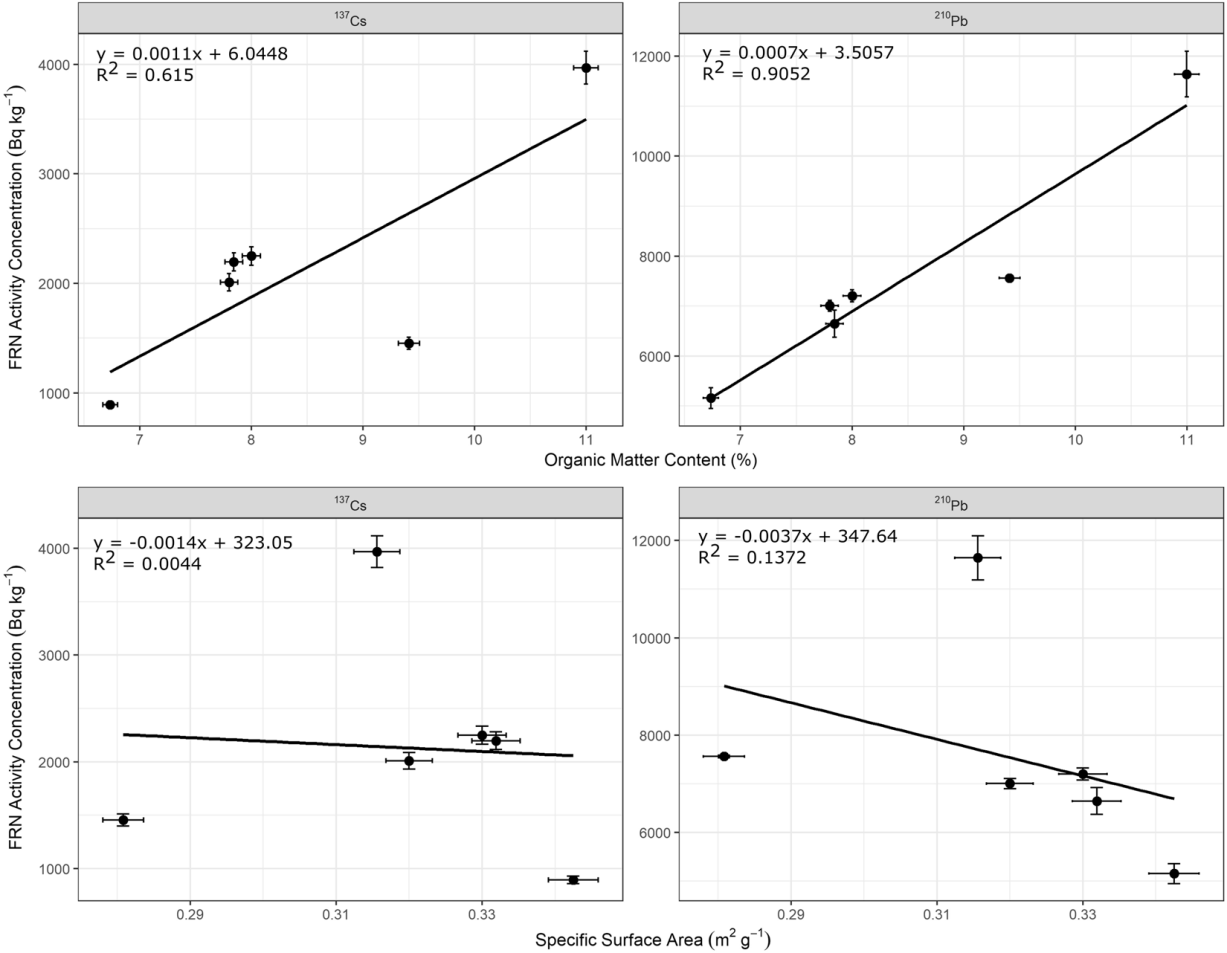
Relations between fallout radionuclide activity concentrations and organic matter content and specific surface area for cryoconite samples from Castle Creek Glacier. Errors bars are ±2 SD.

The cryoconite samples had significant relations (p < 0.05) between activity concentrations and organic matter content for ^137^Cs and ^210^Pb_un_ but not between activity concentrations and specific surface area (Fig. 4). This suggests that organic matter plays a key role in scavenging FRNs and other contaminants. The lack of a relationship between FRN activity concentrations and specific surface areas is somewhat surprising given the documented relation between the two for many soils and sediments^26^. However, it may reflect the similarity in specific surface area values between samples (range 0.28 to 0.34 m^2^ g^−1^) and the higher sorptive capacity of the organic matter and EPS within the cryoconite. The high scavenging associated with organic material is likely to be important in explaining the high enrichment in cryoconite of elements like Hg, Pb and Se relative to local source materials.

The finding that cryoconite is enriched in P relative to surrounding local materials is of note, given that cryoconite microbial communities are likely to be P-limited^6,7^. This suggests that cryoconite communities are scavenging P from meltwater, as P concentrations associated with precipitation, and glacial environments generally, are low. The average value for cryoconite for Castle Creek Glacier (1,180 µg g^−1^; Fig. 3) is lower than that reported for a glacier in Spitsbergen^18^ (2,200 µg g^−1^) but in both cases values are about double that of nearby downstream glaciofluvial sediments.

### Conclusions and downstream implications

Results from Castle Creek Glacier show that FRNs, trace elements, metal(loids), and nutrients, such as P, were elevated in cryoconite. This was likely due to scavenging by fine inorganic particles (i.e. clays) and organic materials (e.g. algae, bacteria and EPS) contained within cryoconite of the meltwater in the ablation zone. Figure 4 indicates that organic material may be the key factor in controlling this scavenging. These findings are similar to other studies that have reported elevated FRNs activity concentrations, and metal(loid)s and nutrients (i.e. P) concentrations in cryoconite on glaciers from the European Alps, Greenland and Svalbard (Table 3), as well as studies that have documented high metal(loid)s concentrations in cryoconite in the Indian Himalaya^19^.

Atmospherically derived contaminants were not particularly high in other clastic materials in the proglacial zone exposed during recent glacier retreat or in actively transported suspended sediments collected from the proglacial stream. This effect was likely due to the low amounts of cryoconite relative to other sources of material such as proglacial deposits, talus and channel banks, which are mobilized during high-flow events in this system^22^. The implications are that enhanced glacial melt and the release of cryoconite associated with climate change may not have significant detrimental effects on aquatic ecosystems and human health in areas located downstream from the immediate proglacial zone, and that any affects will decrease with increasing distance from the glacier snout. However, in situations where there are proglacial lakes and/or lakes within reasonably close proximity to glaciers it is possible that sediment that accumulates on the bottom of the lake could contain elevated levels of contaminants and thus pose a risk to aquatic life in local ecosystems. Equally, if cryoconite represents a larger proportion of the sediment load of a proglacial river, then its effect may be more pronounced. Further studies are required to examine such effects in contrasting settings.

## Methods

### Study site and sample collection

Castle Creek Glacier (53° 2′N, 120° 24′W, unofficial name) is located in the Cariboo Mountains of eastern British Columbia, ~180 km east of the city of Prince George and ~86 km from Mount Robson, the highest mountain in the Canadian Rockies (Fig. 1). The glacier has an area of ~9 km^2^, a length of ~6 km and an elevation range of 1,870 to 2,850 m above sea level. A series of push-moraines and aerial photographs were used^29^ to estimate that the glacier had retreated 886 m between 1946 and 2007. This retreat has exposed new sediments to weathering, erosion and fluvial transport. Several streams drain the glacier snout, most becoming confluent ~0.5 km from the snout. After flowing through the recently exposed proglacial zone, the main proglacial stream passes through a small gorge before flowing ~34 km where it is confluent with the Fraser River, which itself flows from a source near Mount Robson to the Pacific Ocean near Vancouver.

Samples of sediment were collected from 47 locations in the ablation zone and near the glacier snout in summer 2008 and 2011 (Fig. 1). Samples were classified as: (i) cryoconite (n = 6); (ii) subglacial (n = 1); (iii) exposed proglacial sediments near the snout (n = 10); (iv) talus material from nearby slopes (n = 20); and (v) channel bank material from proglacial streams draining the glacier (n = 10). The subglacial sample was collected from inside a large meltwater tunnel at the glacier snout. Samples were collected using a stainless-steel trowel, cleaned between samples, and the samples were air-dried in the field and stored. Bulk samples (n = 10) of suspended sediment (>3 g) were collected in large containers during high-flow events at several locations along the proglacial stream, in additional to small tributary streams, one of which drains the non-glacierized slopes. Fluvial suspended sediments were allowed to settle for 2 days and the supernatant water was removed by syphoning. Suspended sediments were air-dried in the field and stored. All samples were sieved to <63 µm in the laboratory prior to analysis.

### Sample analysis

Analysis of FRNs was carried out at the Consolidated Radio-isotope Facility (ISO 9001) at the University of Plymouth, UK, in 2012 and 2013, following an established methodology^30^. After oven drying, samples were placed into individual sealed 4 mL plastic vials and incubated for a minimum of 24 days to allow establishment of radioactive equilibrium with ^222^Rn. Activity concentrations were determined using an EG&G Ortec well detector (GWL-170-15 S; N-type) with a counting time of at least 24 h. The detector was calibrated using material spiked with certified, traceable mixed radioactive standard 80717-669 supplied by Eckert & Ziegler Analytics (Georgia, USA). All calibration relationships were derived using EG&G GammaVision software. The data were verified by inter-laboratory comparisons with soil IAEA-TEL-2012-03 supplied by the International Atomic Energy Agency (IAEA, Vienna, Austria). The isotopes ^210^Pb (half-life (t_½_) = 22.3 years), ^241^Am (t_½_ = 432.2 years), ^214^Pb (t_½_ = 26.8 mins) and ^137^Cs (t_½_ = 30.2 years) were determined by their gamma emissions at 46.52, 59.54, 295.34 (and 351.99) and 661.6 keV, respectively. Total ^210^Pb was measured and its unsupported component (^210^Pb_un_) was calculated by the subtraction of the radium-226 (^226^Ra; t_½_ = 1600 years) activity, which in turn was measured by the gamma emissions of ^214^Pb. Samples were typically measured for 80,000 s or until the equilibrium point between ^210^Pb and ^214^Pb had been reached. All activity concentration data were decay corrected to 2009. Uncertainties were derived from counting statistics and are reported at 2 sigma. A few samples (n = 10) were analysed for FRNs (^137^Cs and ^210^Pb) at the Environmental Radiochemistry Laboratory (ERL) at the University of Manitoba, Canada, in 2018 following similar procedures described above; the ERL also undergoes inter-laboratory comparison tests with IAEA materials. Checks of select samples between the two laboratories gave differences in activity concentrations of <5%. All values were decay corrected to 2009.

Geochemical elements in the samples were determined in 2012 by a commercial laboratory (ALS Minerals, British Columbia, Canada; ISO 17025 and ISO 9001) after oven drying using inductively-coupled plasma – mass spectrometry (ICP-MS) and inductively-coupled plasma – atomic emission spectrometry (ICP-AES) following aqua regia (HNO_3_ and HCl) digestion. Analytical ranges for the elements are given in Supplementary Table 1.

Some samples were analysed for particle size composition and organic matter content in 2017 and 2018 at the Northern Analytical Laboratory Services at the University of Northern British Columbia (UNBC). Particle size analysis was achieved using a Malvern Mastersizer 3000 (Malvern, UK) laser diffraction system after standard pretreatment with hydrogen peroxide (to remove organic matter) and dispersion with sodium hexametaphosphate. Organic matter was determined by loss-on-ignition at 550 °C.

## Supplementary information

Supplementary material Table 1

## Data Availability

Supplementary Information accompanies this paper. Data are available on request.
