# Supplementary material for: Extreme levels of fallout radionuclides and other contaminants in glacial sediment (cryoconite) and implications for downstream aquatic ecosystems
Source: Sci Rep. 2019 Aug 29;9:12531. doi: 10.1038/s41598-019-48873-z (PMC6715685; doi:10.1038/s41598-019-48873-z)
Supplement: Supplementary file 1 — Supplementary material Table 1 [file 41598_2019_48873_MOESM1_ESM.docx]

**Scientific Reports**

**Supplementary Material to:**

Extreme levels of fallout radionuclides and other contaminants in glacial sediment (cryoconite) and implications for downstream aquatic ecosystems

Philip N. Owens^1^, William H. Blake^2^, Geoffrey E. Millward^2^

^1^Environmental Sciences Program and Quesnel River Research Centre, University of Northern British Columbia, Prince George, British Columbia, V2N4Z9, Canada (Philip.owens@unbc.ca)

^2^School of Geography, Earth and Environmental Sciences, University of Plymouth, Plymouth, Devon, UK (William.blake@plymouth.ac.uk; [g.millward@plymouth.ac.uk](mailto:g.millward@plymouth.ac.uk))

**Supplementary material Table 1 – Analytes and analytical ranges (ppm)**

| Analyte | Range | Analyte | Range |
| --- | --- | --- | --- |
| As | 0.1 – 10,000 | Pb | 0.2 – 10,000 |
| Bi | 0.01 – 10,000 | Rb | 0.1 – 10,000 |
| Ca | 0.01% – 25% | Sb | 0.05 – 10,000 |
| Cd | 0.01 - 1000 | Sn | 0.2 - 500 |
| Cr | 1 – 10,000 | Ti | 0.005% - 10% |
| Cu | 0.2 – 10,000 | W | 0.05 – 10,000 |
| Hg | 0.01 – 10,000 | Zn | 2 – 10,000 |
| P | 10 – 10,000 |  |  |

| \|  \| \| --- \| |  |  |  |  |  |  |
| --- | --- | --- | --- | --- | --- | --- | --- |
